# Genetic variance in Nitric Oxide Synthase and Endothelin Genes among children with and without Endothelial Dysfunction

**DOI:** 10.1186/1479-5876-11-227

**Published:** 2013-09-25

**Authors:** Siriporn Chatsuriyawong, David Gozal, Leila Kheirandish-Gozal, Rakesh Bhattacharjee, Ahamed A Khalyfa, Yang Wang, Hakon Hakonarson, Brendan Keating, Wasana Sukhumsirichart, Abdelnaby Khalyfa

**Affiliations:** 1 Department of Pediatrics, Comer Children’s Hospital, Pritzker School of Medicine, Biological Sciences Division, The University of Chicago, 900 E, 57th Street, KCBD, 4112, Chicago 60637, IL, USA; 2 Department of Biochemistry, Faculty of Medicine, Srinakharinwirot University, Sukhumvit 23, Bangkok 10110, Thailand; 3 Center for Applied Genomics, Children’s Hospital of Philadelphia, University of Pennsylvania, Philadelphia, PA USA

**Keywords:** NOS, EDN, Endothelial, Endothelial dysfunction, SNP, Children

## Abstract

**Background:**

The presence of endothelial dysfunction (ED) constitutes an early risk factor for cardiovascular disease (CVD) in children. Nitric oxide (NO) and endothelin (EDN) are generated in endothelial cells and are critical regulators of vascular function, with ED resulting from an imbalance between these two molecules. We hypothesized that genetic variants in NO synthase and EDN isoforms and its receptors (EDNRA and EDNRB) may account for a proportion of the risk for ED in developing children.

**Methods:**

Consecutive children (ages 5–10 years) were prospectively recruited from the community. Time to peak post-occlusive reperfusion (Tmax) was considered as the indicator of either normal endothelial function (NEF; Tmax < 45 sec) or ED (Tmax ≥ 45 sec). Lipid profiles, high sensitivity C-reactive protein (hsCRP), fasting glucose and insulin were assayed using ELISA. Genomic DNA from peripheral blood was extracted and genotyped for NOS1 (209 SNPs), NOS2 (122 SNPs), NOS3 (50 SNPs), EDN1 (43 SNPs), EDN2 (48 SNPs), EDN3 (14 SNPs), EDNRA (27 SNPs), and EDNRB (23 SNPs) using a custom SNPs array. Linkage disequilibrium was analyzed using Haploview version 4.2 software.

**Results:**

The relative frequencies of SNPs were evaluated in 122 children, 84 with NEF and 38 with ED. The frequencies of NOS1 (11 SNPs), and EDN1 (2 SNPs) were differentially distributed between NEF vs. ED, and no significant differences emerged for all other genes. Significant SNPs for NOS1 and EDN1 SNPs were further validated with RT-PCR.

**Conclusions:**

Genetic variants in the NOS1 and EDN1 genes appear to account for important components of the variance in endothelial function, particularly when concurrent risk factors such as obesity exist. Thus, analysis of genotype-phenotype interactions in children at risk for ED will be critical for more accurate formulation of categorical CVD risk estimates.

## Background

Obesity is one of the world’s greatest public health challenges affecting not only developed, but also developing countries. The presence of obesity has now been conclusively linked to heightened morbidity and mortality through increased risk for many chronic diseases, including type-2 diabetes, hypertension, dyslipidemia, and coronary artery disease [1-4]. The pathogenesis of obesity is multifactorial incorporating both genetics and lifestyle. In children, obesity is associated with increased risk for multifaceted derangements in metabolic and cardiovascular function, including endothelial dysfunction (ED) [5-8]. In general terms, overweight children are more likely to prematurely develop ED, hypertension and type 2 diabetes, an array of conditions that would normally be only found in older obese adults [4]. Recently, we have shown that obesity in children is associated with an increased risk for the development of ED prior to the onset of hypertension [7]. However, not every obese child will develop ED, suggesting that both genetic and environmental factors may play a role. Conversely, a small subset of otherwise healthy children who are not obese may manifest abnormal endothelial function, and such functional phenotype may be determined by genetic variance in endothelial function-related genes.

ED, an early risk marker of cardiovascular disease, refers to a loss of normal homeostatic function in the blood vessels and is characterized by altered vasodilatory and vasoconstrictive functions and inflammatory activity [9]. ED is involved in the development of vascular complications related to dyslipidemia, and cardiovascular disease such as hypertension, coronary artery disease and chronic heart failure [10,11]. A major contributor of ED involves reductions in the amount of bioavailable nitric oxide (NO) in the vasculature [12]. Indeed, NO is an important factor in endothelial functional homeostasis, and also inhibits platelet aggregation, leukocyte adhesion, smooth muscle cell migration and proliferation [13], as well as oxidation of atherogenic low-density lipoprotein [14]. NO is synthesized from the amino acid L-arginine in endothelial cells, as well as many other cell types by three nitric oxide synthase (NOS) isoforms; neuronal (nNOS, NOS1), inducible (iNOS, NOS2), and endothelial (eNOS, NOS3) [15-18]. NOS1 and NOS3 genes are constitutively expressed resulting in a low basal synthesis of NO [19,20], whereas induction of NOS2 expression is regulated by transcription factors such as nuclear factor κB (NF-_K_B) [21]. Several studies have examined the possibility that genetic variants in the genes encoding these enzymes could influence their expression and functional activity, potentially altering the predisposition to cardiovascular disease [22,23]. Accordingly, single nucleotide polymorphisms (SNPs) have been identified in NOS genes, and their association with coronary artery disease, hypertension, and diabetes has been explored [24-26]. To the best of our knowledge, the potential associations between NOS polymorphisms and ED are currently unexplored in children.

The endothelin (EDN) system consists of three endothelin isoforms (EDN1, EDN2 and EDN3), and two receptors (endothelin type A and type B) linked to multiple signaling pathways [27]. Endothelins (EDN1, EDN2 and EDN3) are 21-amino acid peptides that exert their effects through their cognate receptors EDNRA and EDNRB with different degrees of binding affinity. Various SNPs have been identified on EDN genes and EDNRA and EDNRB receptors genes, and have been associated with susceptibility and prognosis of diseases such as heart failure, dilated cardiomyopathy, diabetic retinopathy, and atherosclerosis [28-32]. Again, we are unaware of any published studies on the potential associations between ED and endothelin-related gene variants in children.

Based on aforementioned considerations, we aimed to identify single nucleotide polymorphisms (SNPs) in NOS family (3 isoforms), and EDN family (3 isoforms and their 2 cognate receptors) in order to identify potential associations of these SNPs with ED in children.

## Methods

### Subjects

The study was approved by the University of Louisville Human Research Committee (Louisville, KY, USA), and informed consent was obtained from the legal caregiver of each participant. Consecutive healthy pre-pubertal children (ages 5–10 years) were recruited from the community to investigate endothelial function.

### Anthropometric characteristics

#### Anthropometry

Children were weighed using the InBody 320 scale (Biospace; Cerritos, CA), and height (to 0.1 cm) was measured using a stadiometer (Holtain, Crosswell, UK). The BMI was calculated and the BMI *z*-score was computed using US Centers for Disease Control and Prevention 2000 growth standards (http://www.cdc.gov/growthcharts/) and online software (http://wwwn.cdc.gov/epiinfo/). A BMI *z*-score > 1.65 (> 95th percentile) was considered as fulfilling obesity criteria.

#### Sphygmomanometry

All children had arterial blood pressure measured noninvasively using an automated mercury sphygmomanometer (Welch Allyn; Skaneateles Falls, New York) at the brachial artery, using the appropriate cuff size on the nondominant arm [33]. Systolic BP and diastolic BP indices were calculated by dividing the average systolic and diastolic pressure by the respective 95th percentile for BP using National Heart, Lung and Blood Institute guidelines http://www.nhlbi.nih.gov/guidelines/hypertension/child_tbl.htm), computed for age, sex, and height. Hypertension was defined when the SBPi or DBPi was > 1.

### Endothelial function tests

Endothelial function was assessed using a modified hyperemic test after cuff-induced occlusion of the radial and ulnar arteries by placing the cuff over the wrist. All testing was performed in the morning after an overnight fast. A laser Doppler sensor (Periflux 5000 System integrated with the PF 5050 Pressure Unit, Perimed, Järfälla, Sweden) was applied over the volar aspect of the hand at the 1st finger distal metacarpal surface and the hand was gently immobilized. This site was chosen as an area in order to minimize the effects of motion artifact, and was also found to have a density of skin capillary blood flow that was of appropriate magnitude for detection. Children lay supine with the head of the bed elevated 45°. Once cutaneous blood flow over the area became stable, the pressure within an inflatable cuff placed at the forearm and connected to a computer- controlled manometer was raised to 200 mmHg for 60 seconds during which blood flow was reduced to undetectable levels. An occlusion time of 60 seconds was chosen in order to minimize discomfort for the child and thus prevent motion and invalidation of the test. Furthermore, we used a computer-controlled pressure release to allow for consistent deflation times. The cuff was rapidly deflated, and the laser Doppler measured hyperemic responses. The maneuver was repeated twice within 10 min with at least 2 min separating both trials to ensure a return to baseline perfusion. The average of both maneuvers was then computed for subsequent analyses, and demonstrated excellent reproducibility [34]. Commercially available software (Perimed, Järfälla, Sweden) allowed for unbiased estimates of the time to peak regional blood flow response post-occlusion release (Tmax), which is considered representative of the hyperemic response and an index of endothelial function [35]. As previously described, we defined abnormal endothelial function as a Tmax cutoff value of ≥45 seconds [34].

### Plasma assays

Fasting blood samples were drawn from the subjects and immediately centrifuged in a cooled centrifuge and plasma was frozen at −80°C until assay, usually within <2 weeks from collection. Plasma insulin levels were measured using a commercially available radioimmunoassay kit (Coat-A Count Insulin; Diagnostic Products Inc). This method has a detection level of 1.2 μIU/mL and exhibits linear behavior up to 350 μIU/mL, with intra-assay and inter-assay coefficients of variability of 3.1% and 4.9%, respectively. Plasma glucose level was measured using a commercial kit based on the hexokinase-glucose-6-phosphate dehydrogenase method (Flex Reagent Cartridges; Dade Behring, Newark, DE). Insulin resistance was assessed using the homeostasis model assessment (HOMA) equation (fasting insulin × fasting glucose/22.5) [36]. Plasma hsCRP levels were measured within 2–3 hours after collection using the Flex reagent Cartridge (Date Behring, Newark, DE). This method has a detection level of 0.05 mg/dl, and exhibits linear behavior up to 255 mg/dl, with intra-assay and inter-assay coefficients of variability of 9% and 18% respectively for hsCRP. Serum lipids including total cholesterol, high-density lipoprotein (HDL) cholesterol, calculated low-density lipoprotein cholesterol (LDL), and triglycerides (TG) were also assessed using Flex Reagent Cartridges (Dade Behring). To ensure consistency and to prevent protein degradation, particular care was taken to standardize all steps of plasma sample processing and to minimize thawing more than once for each aliquot.

### DNA extraction

Peripheral blood samples were collected in vacutainer tubes containing EDTA (Becton Dickinson, Franklin Lakes, NJ, USA). Genomic DNAs of children were extracted using QIAmp DNA blood kit (Qiagen, Valencia, CA, USA). The concentration and quality of the DNA were determined using a ND-1000 Spectrophotometer (Nanodrop Technologies, Wilmington, DE, USA). The purity of the DNA were determined by calculating the ratio of absorbance at 260/280 nm, and all DNA samples had a ratio of 1.8–1.9. The precise length of genomic DNA was determined by gel electrophoresis using 1% agarose gel. All the purified samples were stored at −80°C until further analyses.

### Custom cardiovascular gene SNP array

The IBC array was developed using SNP and linkage disequilibrium information from the HapMap as well as resequencing data from Seattle SNPs and National Institute of Environmental Health Sciences (NIEHS) SNPs [37]. Briefly, the IBC array contains about 50,000 SNPS from genetic diversity across approximately 2100 genes related to cardiovascular, inflammatory, hemostasis/coagulation, and metabolic phenotypes and pathways. Among those genes, we selected the NOS genes which include NOS1 (209 SNPs), NOS2 (122 SNPs) and NOS3 (50 SNPs). Furthermore we selected the EDN and EDN receptor genes family which includes EDN1 (43 SNPs), EDN2 (48 SNPs), EDN3 (14SNPs), EDNRA (27 SNPs) and EDNRB (23 SNPs). SNPs were clustered into genotypes with the Illumina Beadstudio software and subjected to quality-control filters at the sample and SNP levels separately within each cohort. Samples were excluded for individual call rates <90%, gender mismatch, and duplicate discordance. SNPs were removed for call rates <95% or Hardy-Weinberg Equilibrium p < 10^−7^ in controls from each cohort (regardless of ethnicity). Because of the low-frequency SNPs included in the design and the aim to capture low-frequency variants of large effect across the large dataset, we filtered only on minor allele frequency (MAF) < 0.005.

### Validation of SNP array using real-time PCR

SNPs that showed statistically significant differences between children with ED and without ED were further validated using TaqMan real-time PCR (Applied Biosystems, Inc.). Two fluorogenic minor groove binder probes were used for each locus using the dyes 6-carboxyfluorescein (FAM; excitation, 494 nm) and VIC (excitation, 538 nm) which are easily differentiated in RT-PCR system. The reaction mixture (25 μl total volume per single well reaction) containing 12.5 μl of TaqMan 2X universal master mix (Applied Biosystems, CA), 1.25 μl of each primer, 10.25 μl of RNase- and DNase-free water (Ambion, Austin, TX), and 1 μl of DNA template. Each sample was duplicated in two wells of a 96 well-plate (Applied Biosystems, CA). DNase-free water used as negative control was included in each assay run. Protocol consisted of 2 min at 50°C, 10 min at 95°C, and 42 cycles of 95°C for 15 sec and 60°C for 1 min. Initially, the SNP assay was set up using SDS, version 2.1, software (Applied Biosystems, CA) as an absolute quantification assay, but after assay completion the plate was read using the allelic discrimination settings. Post-assay analysis was performed using the SDS software. The goodness-of-fit test for Hardy–Weinberg equilibrium (HWE) was performed after analysis using the equation p^2^ + q^2^ + 2pq = 1, where p and q represent the wild-type and variant allele of a gene.

### Total RNA isolation

Fasting peripheral blood samples were drawn from children within the first hour after awakening and collected in PAXgene Blood RNA tubes (Becton Dickinson, UK). Total RNA was isolated using PAXgene Blood RNA Kit and treated with DNase I (QIAGEN, CA), according to the manufacturer’s protocol. The RNA quantity and integrity were determined using a Nanodrop Spectrophotometer and Agilent 2100 Bioanalyzer Nano 6000 LabChip assay (Agilent Technologies).

### Gene expression using qRT-PCR

Quantitative real time RT-PCR (qRT-PCR) analyses were performed using ABI 7500 (Applied Biosystems, Foster City, CA). cDNA synthesis was performed using a High-Capacity cDNA Archive Kit (Applied Biosystems, Foster City, CA). Five hundred nanograms (500 ng) of total RNA from NEF and ED samples were used to generate cDNA templates for RT-PCR. The TaqMan® Master Mix Reagent Kit (Applied Biosystems, Foster City, CA) was used to amplify and quantify each transcript of interest in 25 μl reactions. Various negative controls were included in the PCR reaction to ensure specific amplification. Triplicate PCR reactions were performed in 96-well plates for each gene in parallel with the 18S rRNA. The steps involved in the reaction program included: the initial step of 2 minutes at 50°C; denaturation at 95°C for 10 min, followed by 45 thermal cycles of denaturation (15 seconds at 95°C) and elongation (1 min at 60°C). The expression values were obtained from the cycle number (*Ct* value) using the Biosystems analysis software. The threshold cycle (*C*_T_) values were averaged from each reaction, and each gene was normalized to the 18S rRNA level. All the genes of interest and 18S rRNA were performed in triplicates to determine the Ct-diff. These Ct values were averaged and the difference between the 18S Ct (Avg) and the gene of interest Ct (Avg) was calculated (Ct-diff). The relative expression of the gene of interest was analyzed using the 2^-ΔΔCT^ method [38]. Quantitative results are expressed as the mean ± standard deviation (SD). Statistical significance was evaluated by the Student’s *t*-test.

### Statistical analysis

All analyses were conducted using SPSS software (version 19.0; SPPS Inc., Chicago, Ill.), and data are presented as mean ± SD. The association analysis was assessed by using Pearson’s chi-square test implemented in SPSS. A *P*-value < 0.05 was considered statistically significant for all analyses. Odds ratio and 95% confidence interval were calculated for the minor allele of each SNP. We performed these analyses under the a priori assumption that since candidate-gene studies lack power to detect weak genetic risk effects of common variants, any findings from the current analyses will require cautious interpretation. Indeed, to achieve a power of >80% in the detection of a modest genetic risk (e.g., odds ratio = 1.2) for any SNP of interest with a known prevalence of 10% in the population, a sample size of more than 10,000 subjects would be needed [39,40]. The Haploview version 4.2 software (http://www.borad.mit.edu/mpg/haploview) was used to analyze the linkage disequilibrium structure, calculating D’ to define haplotype block [41] and to estimate haplotype frequencies. Additionally, pair-wise linkage disequilibrium (LD) among the SNPs was examined using Lewontin’s standardized coefficient D’ and LD coefficient r^2^[42], and haplotype blocks were defined according to the method of Garbriel et al. [41] in Haploview 4.2 with default settings. Haplotypes within these blocks were estimated using the estimation of maximization algorithm [43].

## Results

### Cohort phenotype

**Figure 1:**
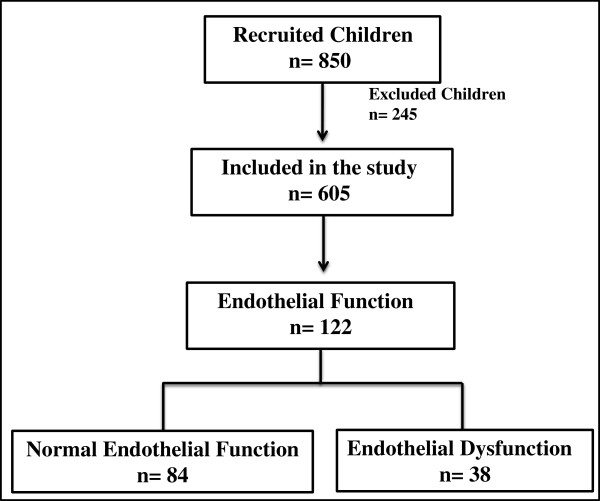
**Schematic diagram illustrating the recruitment process.** Children were matched for age, gender and ethnicity. Children were excluded from the study, if they had any chronic medical conditions such as known genetic syndromes, severe asthma or allergies, or if they were on any chronic medications.

Of a potential total of 850 subjects, > 600 subjects were recruited from the community, and of these, 122 children were randomly selected and their endothelial function was tested (Figure 1). Approximately 245 subjects were excluded from the study due to chronic medical conditions such as Down syndrome, craniofacial or known genetic syndromes, a known episode of infection in the eight weeks preceding the sleep study, asthma or allergies receiving specific therapy (desensitization, leukotriene inhibitors, steroids (topical or systemic). Eighty four children were found to have NEF and 38 had evidence of ED as defined by their individual Tmax values. The demographic characteristics of these subjects are shown in Table 1. The BMI- z score and the proportion of obese children were significantly higher (*P*-value ≤ 0.0002) in children with ED. However, there were no differences in either systolic or diastolic blood pressures between both groups. The metabolic data for the 2 groups are shown in Table 1. Only serum triglyceride levels (TG) were significantly higher in children with ED (*P*-value ≤ 0.04), with both groups exhibiting similar fasting serum TC, HDL and LDL cholesterol. Similarly, fasting glucose levels were similar, but plasma insulin concentrations were higher in ED children (*P*-value ≤0.05). Serum levels of high sensitivity C-reactive protein (hsCRP) were also similar (Table 1).

**Table 1:** Demographic characteristics and metabolic data in children with and without endothelial dysfunction

**Variables**	**Endothelial function** **(n = 84)**	**Endothelial dysfunction** **(n = 38)**	***P*-value**
Age (years)	7.60 ± 1.22	8.05 ± 1.32	0.14
Gender (% male)	70.2	63.2	0.16
Ethnicity			
White Caucasian %	77.9	74.3	0.19
African American %	22.1	25.7	0.22
^1^BMI z-score	0.99 ± 1.26	1.75 ± 0.98	0.0002
^2^SBP (mmHg)	105.35 ± 10.84	105.31 ± 11.12	0.50
^3^DBP (mmHg)	62.60 ± 7.57	62.11 ± 7.70	0.40
^4^Tmax (sec)	27.47 ± 10.65	61.97 ± 16.92	0.0001
^5^TG (mg/dl)	71.69 ± 36.42	88.97 ± 52.80	0.04
^6^TC (mg/dl)	160.60 ± 25.99	166.72 ± 27.12	0.13
^7^HDL-C (mg/dl)	52.77 ± 10.46	49.67 ± 8.66	0.05
^8^LDL-C (mg/dl)	93.19 ± 22.11	99.22 ± 23.68	0.10
Glucose (mg/dl)	78.72 ± 16.48	80.59 ± 13.63	0.28
Insulin (mg/dl)	6.44 ± 5.34	8.77 ± 6.53	0.05
^9^HOMA-IR	1.12 ± 1.21	1.27 ± 1.54	0.29
^10^CRP (mg/dl)	1.24 ± 3.51	1.32 ± 1.76	0.44

^1^BMI Z-score (body mass index-z-score; ^2^SBP (systolic blood pressure); ^3^DBP (diastolic blood pressure); ^4^Tmax (time of maximum perfusion); ^5^TG (Triglycerides); ^6^TC (total cholesterol); ^7^HDL-C (high-density lipoprotein)-cholesterol; ^8^LDL-C (low-density lipoprotein)-cholesterol; ^9^HOMA-IR (homeostatic model assessment-insulin resistance); and ^10^CRP (C-reactive protein).

### NOS genes

As a preamble, all the SNPs tested in this study were in Hardy-Weinberg equilibrium. From a total of 381 SNPs assayed for the 3 NOS genes, 11 SNPs in NOS1 exhibited statistically significant differences in their frequencies among children with and without ED both in the univariate analysis and after correction for multiple comparisons (Table 2). The *P*-value in Table 2 represents the significance of the allele frequency for each SNP. In addition, the odds ratio (OR) and the confidence intervals of 95% (CI) for all SNPs are shown in Table 2. For example, in addition, to the *P*-value for the allelic frequency, if a SNP presented in Table 2 is higher than 1.0 that indicates this SNPs is significantly associated with ED in the children. Furthermore, all SNPs in Table 2 have a higher OR and CI than 1.0 with the exception of one SNP (rs9658255), indicating that these SNPs are most likely to be associated with ED. No differences emerged for either NOS2 or NOS3 SNPs.

**Table 2:** Distributions of allele and genotype frequencies of NOS1 SNPs in children with and without endothelial dysfunction

**SNP**	**Allele**	**Endothelial function**		**Endothelial dysfunction**		***P-*value**	**OR**	**CI 95%**
		**n = 84**		**n = 38**				
rs6490121	A/G	n	%	n	%			
	AA	31	37	20	53	0.046	5.26	1.16-23.89
	GA	34	40	16	42			
	GG	19	23	2	5			
	Allele A	96	57	56	74			
	Allele G	72	43	20	26			
rs2293052	C/T	n	%	n	%			
	CC	53	63	16	42	0.016	1.76	0.46-6.71
	TC	20	24	19	50			
	TT	11	13	3	8			
	Allele C	126	75	51	67			
	Allele T	42	25	25	33			
rs3825102	A/C	n	%	n	%			
	AA	11	13	2	5	0.001	2.97	1.33-6.62
	AC	22	26	23	61			
	CC	51	61	13	34			
	Allele A	44	26	27	36			
	Allele C	124	74	49	64			
rs4767529	C/G	n	%	n	%			
	CC	44	52	12	32	0.049	1.28	0.38-4.31
	GC	29	35	22	58			
	GG	11	13	4	10			
	Allele C	117	70	46	61			
	Allele G	51	30	30	39			
rs561712	A/G	n	%	n	%			
	AA	13	15	4	10	0.037	2.34	1.03-5.32
	AG	30	36	23	61			
	GG	41	49	11	29			
	Allele A	56	33	31	41			
	Allele G	112	67	45	59			
rs549098	C/T	n	%	n	%			
	CC	43	51	11	29	0.032	1.21	0.40-3.67
	TC	28	33	22	58			
	TT	13	16	5	13			
	Allele C	114	68	44	58			
	Allele T	54	32	32	42			
rs567581	A/G	n	%	n	%			
	AA	13	16	5	13	0.022	2.70	1.19-6.14
	AG	27	32	22	58			
	GG	44	52	11	29			
	Allele A	53	32	32	42			
	Allele G	115	68	44	58			
rs483589	A/G	n	%	n	%			
	AA	11	13	11	29	0.008	3.58	1.47-8.70
	AG	32	38	19	50			
	GG	41	49	8	21			
	Allele A	54	32	41	54			
	Allele G	114	68	35	46			
rs693534	A/G	n	%	n	%			
	AA	11	13	13	34	0.011	2.67	1.15-6.18
	AG	32	38	15	40			
	GG	41	49	10	26			
	Allele A	54	32	41	54			
	Allele G	114	68	35	46			
rs9658255	C/G	n	%	n	%			
	CC	41	49	10	26	0.029	0.37	0.14-0.95
	GC	32	38	17	45			
	GG	11	13	11	29			
	Allele C	114	68	37	49			
	Allele G	54	32	39	51			
rs1879417	C/T	n	%	n	%			
	CC	14	17	14	37	0.02	2.73	1.07-6.91
	CT	38	45	17	45			
	TT	32	38	7	18			
	Allele C	66	39	45	59			
	Allele T	102	61	31	41			

**Figure 2:**
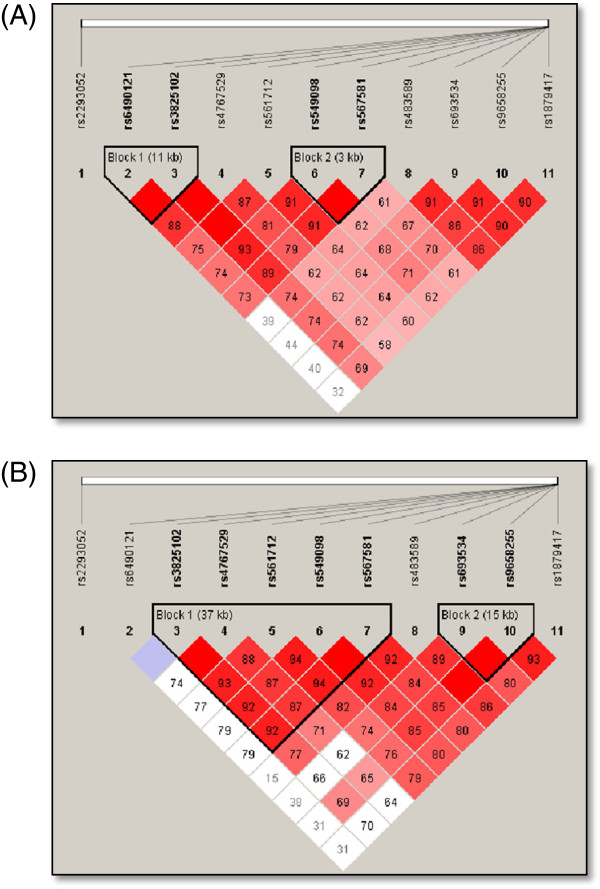
**Pairwise linkage disequilibrium (LD) structure and 11 SNPs of the NOS1 gene.** Panel **(A)** represents children with normal endothelial function (NEF), and Panel **(B)** represents children with endothelial dysfunction (ED). The plot was generated using Haploview 4.2 with D’ Color Scheme (D’ = 0, D’ < 1 and D’ = 1 shown by white, shades of pink and red (respectively) and pairwise r^2^ values shown in diamonds. The value within each diamond represents the pair-wise LD (correlation, measured as D’) between the two SNPs defined by the top left and the top right of the diamond. Solid lines represent SNPs that were used in the haplotype analysis, and are part of the haplotype from SNP block whereas dashed lines represent SNPs that were used in the analysis, but were not part of the haplotype.

**Figure 3:**
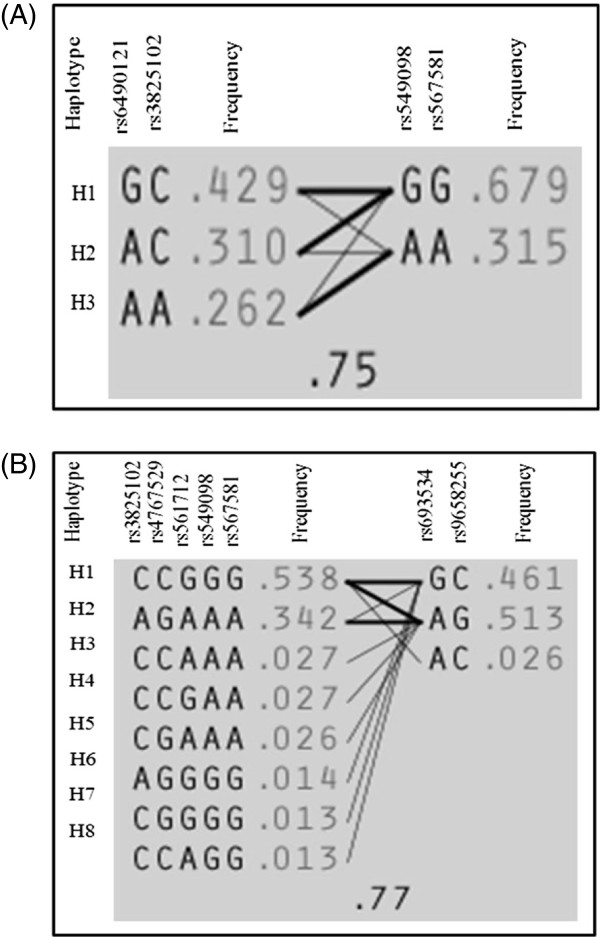
**Haplotype frequencies in children with and without endothelial dysfunction.** Panel **(A)** represents haplotype for children with normal endothelial function (NEF), and Panel **(B)** represents haplotype for children with endothelial dysfunction (ED).

**Figure 4:**
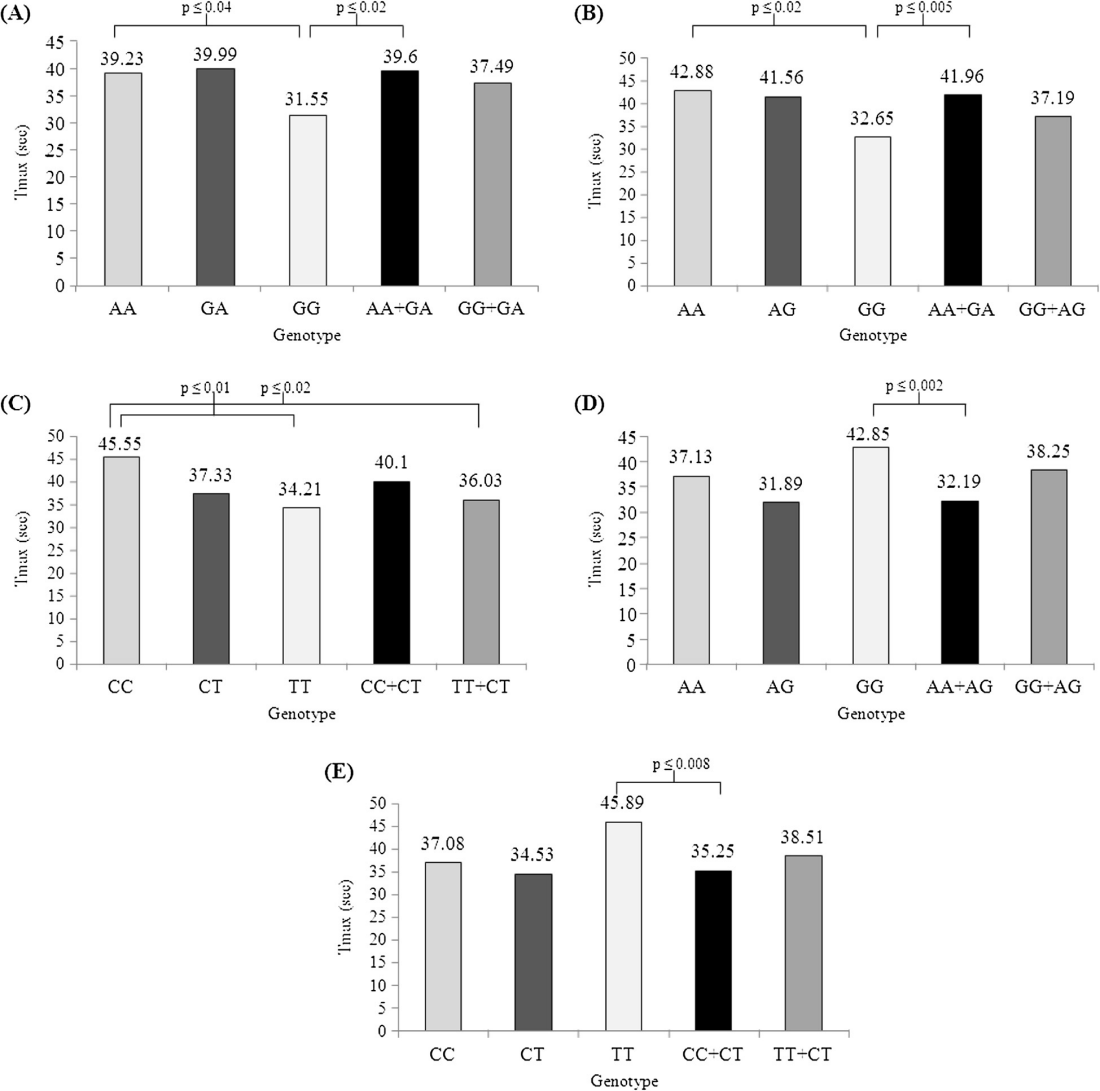
**Comparisons of allele frequencies (genotype) and Tmax values (phenotype) for the NOS1 and EDN1 significant SNPs.** Panel **(A)** represents NOS1 (rs6490121 A/G), Panel **(B)** represents NOS1 (rs483589 A/G), Panel **(C)** represents NOS1 (rs1879417 C/T), Panel **(D)** represents EDN1 (rs1476046 A/G) and Panel **(E)** represents EDN1 (rs4714384 C/T), respectively.

Linkage disequilibrium (LD) analysis of the 11 SNPs in the NOS1 gene was assessed for both NEF and ED subjects. In NEF subjects, two haplotype blocks emerged, and are outlined in black triangular regions in Figure 2 (Panel A). In ED subjects, the haplotype showed the presence of 2 blocks (Figure 2, Panel B). The haplotype of these blocks and their frequencies in NEF and ED are shown in Figure 3, Panels A and B, respectively. Taken together, the patterns of LD and haplotype frequencies differed between NEF and ED, suggesting that some of these SNPs may contribute to ED risk. Therefore, allelic data were analyzed in conjunction with Tmax values among ED and NEF subjects for the significant NOS1 SNPs as shown in Table 3. Of the 11 significant SNPs of the NOS1 gene, there were only 3 SNPs (rs6490121, rs483589, rs1879417) that showed significantly different Tmax based on the genotype. We found that the A allele in rs6490121 (A/G) and rs483589 (A/G) had significantly higher Tmax than the G allele (Figure 3, Panels A and B), while the C allele in rs1879417 (C/T) had significantly higher Tmax values compared to the T allele (Figure 4, Panel C).

**Table 3:** Summary of genotypes-phenotypes for NOS1 SNPs that were classified based on Tmax values in all children

**Number of significant SNPs**	**SNP**	**Allele frequencies**				
1	rs6490121	*AA	GA	*GG	*AA + GA	GG + GA
	(A/G)	(n = 51)	(n = 50)	(n = 21)	(n = 101)	(n = 71)
	Tmax	*39.23 ± 22.10	39.99 ± 21.14	*31.55 ± 13.57	39.60 ± 21.53	37.49 ± 19.51
2	rs2293052	CC	TC	TT	CC + TC	TT + TC
	(C/T)	(n = 69)	(n = 39)	(n = 14)	(n = 108)	(n = 53)
	Tmax	36.50 ± 19.02	42.13 ± 23.15	35.80 ± 20.22	38.53 ± 20.68	40.45 ± 22.40
3	rs3825102	AA	AC	CC	AA + AC	CC + AC
	(A/C)	(n = 13)	(n = 45)	(n = 64)	(n = 58)	(n = 109)
	Tmax	35.02 ± 20.82	43.95 ± 24.46	34.84 ± 16.56	41.95 ± 23.82	38.60 ± 20.59
4	rs4767529	CC	GC	GG	CC + GC	GG + GC
	(C/G)	(n = 56)	(n = 51)	(n = 15)	(n = 107)	(n = 66)
	Tmax	35.66 ± 16.94	41.43 ± 24.04	36.84 ± 19.90	38.41 ± 20.74	40.39 ± 23.10
5	rs561712	AA	AG	GG	AA + AG	GG + AG
	(A/G)	(n = 17)	(n = 53)	(n = 52)	(n = 70)	(n = 105)
	Tmax	35.26 ± 19.75	41.67 ± 23.43	35.66 ± 17.29	40.12 ± 22.62	38.70 ± 20.74
6	rs549098	CC	TC	TT	CC + TC	TT + TC
	(C/T)	(n = 54)	(n = 50)	(n = 18)	(n = 104)	(n = 68)
	Tmax	35.21 ± 16.44	42.04 ± 24.54	36.61 ± 18.86	38.50 ± 20.91	40.60 ± 23.16
7	rs567581	AA	AG	GG	AA + AG	GG + AG
	(A/G)	(n = 18)	(n = 49)	(n = 55)	(n = 67)	(n = 104)
	Tmax	36.61 ± 18.86	42.26 ± 24.75	35.14 ± 16.30	40.74 ± 23.31	38.50 ± 20.91
8	rs483589	*AA	AG	*GG	*AA + AG	GG + AG
	(A/G)	(n = 22)	(n = 51)	(n = 49)	(n = 73)	(n = 100)
	Tmax	*42.88 ± 19.56	41.56 ± 23.18	*32.65 ± 16.88	*41.96 ± 22.02	37.19 ± 20.73
9	rs693534	AA	AG	GG	AA + AG	GG + AG
	(A/G)	(n = 24)	(n = 47)	(n = 51)	(n = 71)	(n = 98)
	Tmax	43.82 ± 19.41	38.19 ± 20.74	35.61 ± 20.79	40.09 ± 20.34	36.85 ± 20.70
10	rs9658255	CC	GC	GG	CC + GC	GG + GC
	(C/G)	(n = 51)	(n = 49)	(n = 22)	(n = 100)	(n = 71)
	Tmax	35.22 ± 20.80	39.53 ± 20.53	42.24 ± 19.98	37.33 ± 20.68	40.37 ± 20.26
11	rs1879417	*CC	CT	*TT	CC + CT	*TT + CT
	(C/T)	(n = 28)	(n = 55)	(n = 39)	(n = 83)	(n = 94)
	Tmax	*45.55 ± 19.68	37.33 ± 21.45	*34.21 ± 18.97	40.10 ± 21.11	*36.03 ± 20.41

*Indicates significant alleles.

### Endothelin related genes

From a total of 155 available SNPs, there were only 2 SNPs in the EDN1 gene whose frequencies differed among children with and without ED. The allele and genotype frequencies of EDN1 SNPs are shown in Table 4. These 2 EDN1 SNPs were analyzed in conjunction with corresponding Tmax values among ED and NEF subjects. We found that both of these SNPs in EDN1 (rs1476046, rs4714384) showed significant differences in Tmax based on genotype (Table 5). The G allele in rs1476046 (A/G) had a significantly higher Tmax than the A allele (Figure 4, Panel D), and the T allele in rs4714384 (C/T) had a significantly higher Tmax than the C allele (Figure 4, Panel E).

**Table 4:** Distributions of allele and genotype frequencies for EDN1 SNPs in children with and without endothelial dysfunction

**SNP**	**Allele**	**Endothelial function**		**Endothelial dysfunction**		***P-*value**	**OR**	**CI 95%**
		**n = 84**		**n = 38**				
rs1476046	A/G	n	%	n	%			
	AA	2	2	1	3	0.032	0.34	0.15-0.79
	AG	41	49	9	24			
	GG	41	49	28	73			
	Allele A	45	27	11	15			
	Allele G	123	73	65	85			
rs4714384	C/T	n	%	n	%			
	CC	22	26	3	8	0.016	0.38	0.16-0.86
	CT	44	52	19	50			
	TT	18	22	16	42			
	Allele C	88	52	25	33			
	Allele T	80	48	51	67			

**Table 5:** Summary of genotypes-phenotypes for EDN1 SNPs that were classified based on Tmax values in all children

**Number of significant SNPs**	**SNP**	**Allele frequencies**				
1	EDN1	AA	AG	*GG	*AA + AG	GG + AG
	rs1476046(A/G)	(n = 3)	(n = 50)	(n = 69)	(n = 53)	(n = 119)
	Tmax	37.13 ± 18.47	31.89 ± 19.20	*42.85 ± 20.62	*32.19 ± 19.03	38.25 ± 20.68
2	EDN1	CC	CT	*TT	*CC + CT	TT + CT
	rs4714384 (C/T)	(n = 25)	(n = 63)	(n = 34)	(n = 88)	(n = 97)
	Tmax	37.08 ± 23.65	34.53 ± 17.56	*45.89 ± 21.82	*35.25 ± 19.38	38.51 ± 19.81

*Indicates significant alleles.

### qRT-PCR SNP assay validation

To confirm the SNP array-based findings, we performed qRT-PCR using TaqMan assays with specific primers for some of the SNPs of interest, i.e., those with significantly different frequencies in their allelic genotype frequencies (Additional file 1: Table S1), as well as some of those with significantly different genotype-phenotype allelic frequencies (Additional file 1: Table S1). The concordance findings between array and real-time PCR for allele and genotype frequencies of NOS1 and EDN1 SNPs in children with and without ED are shown in Additional file 2: Table S2. The concordance for both SNPs in the NOS1 gene (rs3825102, rs483589) was 53.21%, and 61.47%, respectively and 49% for EDN1 SNPS (rs1476046, rs4714384). The summary of the validated SNPs data for both NOS1 and EDN1 genes between SNPs array and qRT-PCR is shown in Additional file 3: Table S3.

### qRT-PCR gene expression

**Figure 5:**
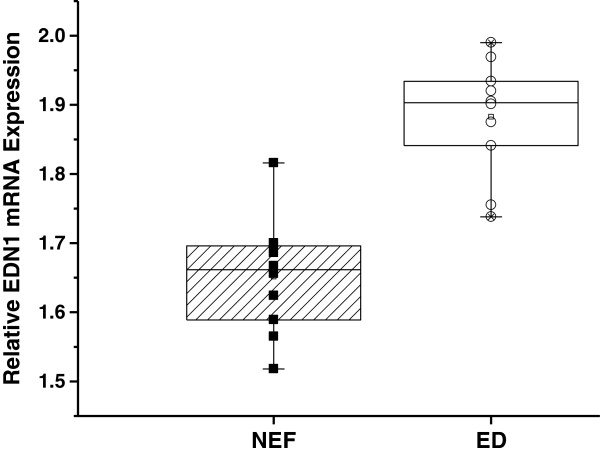
**Real-time PCR analysis for EDN1 gene expression in children with normal endothelial function (NEF) and endothelial dysfunction (ED).** Total RNA was isolated from peripheral blood and transcribed into cDNA. The data presented as mRNA expression levels normalized to 18 s ribosomal RNA concentrations. Results are presented as individual values and as boxplots (*P*-value < 0.0004).

Next we asked how the SNPs that putatively affected endothelial function, specifically, their association with gene expression. We selected a total of 20 matched subjects, 10 with endothelial dysfunction (ED), and 10 with normal endothelial function (NEF), and performed gene expression analysis using qRT-PCR. A significant increase in EDN1 gene expression in the peripheral blood of children with ED emerged compared to NEF as illustrated in Figure 5 (*P*-value 0.0004).

## Discussion

In this study, we report on the associations between the frequency of the 3 NOS genes, and 3 genes of the EDN family, along with its two cognate receptors in a carefully phenotyped cohort of pre-pubertal otherwise healthy community children with and without ED. Not surprisingly, an increased risk of obesity and of increased serum TG levels was present in children with ED. The latter may underlie previously reported lower nitric oxide levels in the context of elevated TG [44]. In addition, the relative frequencies of only a selected few NOS1 and EDN1 gene polymorphisms differed among children with ED, and assessment of Tmax based on allelic variants further confirmed a higher risk of increased Tmax among those children harboring the polymorphic allele.

Before we discuss the potential significance of our findings, some of the strengths and limitations of the study should be mentioned. First, the use of the laser Doppler technique for assessment of vascular responses following cuff-induced arterial occlusion not only allows reproducible determinations of the kinetics of post-ischemic reperfusion, but also provides an accurate reporter of NO-mediated physiological recruitment of the microvasculature [34,45]. The selection of the 60-seconds occlusion time, rather than the more extensively used 5 minutes in adults, was necessary based on a vast set of preliminary experiments aimed at preventing motion-induced artifacts, and thus preserving the validity of the tests. In addition, all Tmax assessments were conducted in the fasting state at the same time of the day to minimize potential confounders introduced by differences in meal content and timing relative to the testing, and also to ensure that circadian variation in endothelial function would not play a role. An additional strength of the study was the inclusion of a priori healthy children from the community. In addition, we excluded children with a variety of diagnoses that can be associated with endothelial dysfunction [46]. Two important limitations of this study include the relatively small size of the cohort of children studied which could hamper statistical power, and the absence of endothelin plasma level measurements. However, these data may be incorporated into multicenter-based meta-analyses that will undoubtedly enhance the validity of the present putative findings. Finally, in the context of our qRT-pCR validation efforts using TaqMan SNP assays, we found substantial discrepancies between the two methods (i.e., SNP arrays and qRT-PCR), even if the significance of the findings remained unaltered. The concordance between the array data and qRT-PCR revolved around only 50-60%. This was surprising considering that the concordance between gene expression arrays and qRT-PCR is remarkably high. It therefore appears that this may not be case for SNP arrays, and the discordant findings necessarily mandate that future studies should require an additional validation step of the array findings using alternative methodologies, an approach that has not been pursued to date while using the same 50 k SNP array as used herein or other commercially available arrays [47-50].

Obesity is a prominent risk factor for the development of cardiovascular disease, diabetes mellitus, dyslipidemia, hypertension, and their constellation as the metabolic syndrome in both adults and children [51-55]. We and others have previously shown that obese children are at higher risk for ED and that both the vasodilatory and vasoconstrictive responses are important. Interestingly, in a recent study Tounian and colleagues reported their analyses of a cohort of 232 severely obese children in whom 12 gene polymorphisms representing selected variants previously associated with vascular ED in adults were examined [56]. These investigators did not find any significant association between flow-mediated vasodilation and any of the 12 SNPs studied. Based on our current findings, we propose that the increased risk for ED in obesity may be explained at least in part by the presence of gene variants in NOS and EDN.

Several studies have investigated the NOS genes family SNPs in relation to different diseases, primarily involving the cardiovascular system [57-60]. In the present study, we found 11 SNPs in the NOS1 gene showing significant differences between children with and without ED. In addition, out of these 11 SNPs, we found 3 SNPs (rs6490121, rs483589, and rs1879417) that were significantly associated with altered Tmax values in children, suggesting that these SNPs carry biological significance. However, no significant differences or associations among SNPs in either NOS2 or NOS3 genes were found.

Similar to studies on NOS gene SNPs, several studies suggest that some of the EDN1 SNPs may be associated with hypertension. For example, EDN1 SNP (rs5370) Lys198Asn (G/T), interactions between BMI and blood pressure emerged in overweight/obese subjects [61-64]. This non-synonymous SNP has already been associated with heart failure and hypertension [61,65]. Furthermore, in rheumatoid arthritis patients who carry the T-T haplotype in EDN1 SNPs (rs1800541 and rs5370), increased systolic blood pressure and high EDN1 plasma levels emerged [66]. In this study, we identified 2 significant SNPs (rs1476046, rs4714384) in the EDN1 gene, and both of these SNPs showed significant differences in Tmax values, adding corroborative evidence to the assumption that these SNPs have biological significance. However, no significant differences emerged in the allelic frequencies in EDN2, EDN3, EDNRA, and EDNRB.

In summary, significant associations of polymorphisms in NOS and EDN genes are apparent among young children with ED. Of note, EDN1 gene expression appears to be increased in the context of ED. Accordingly; further functional studies are needed to clarify the role of variants of NOS and EDN genes in the pathophysiology of cardiovascular disease.

## Competing interests

The authors declare that they have no competing interests.

## Authors’ contributions

SC performed data analysis, and qRT-PCR validation, and help in writing in method section, DG provided the conceptual design of the project, participated in the data analysis and editing final version of the manuscript, L K-G participated in recruited subjects, data analysis and sleep studies, R B participated in recruited subjects, AAK participated in genotypic-phenotypic polymorphisms for pairwise linkage disequilibrium structure and haplotype frequencies using Haploview software, YW reviewed data, BK and HH contributed to genomic polymorphism analyses, WS participated in general discussion and review data, and AK carried data analysis, overall the project and SNPs analysis, writing and editing manuscript. All authors read and approved the final manuscript.

## Funding sources

DG is supported by National Institutes of Health grants HL-065270 and HL-086662. SC is the recipient of the Royal Golden Jubilee Ph. D. Program Award from the Thailand Research Fund (PHD/0138/2552).

## Supplementary Material

Additional file 1: Table S1 List of single nucleotide polymorphisms (SNPs) of NOS1 and EDN1 genes. Click here for file

Additional file 2: Table S2 Distributions of allele and genotype frequencies of NOS1 and EDN1 SNPs in children with and without endothelial dysfunction compare between array and real-time PCR. Click here for file

Additional file 3: Table S3 Comparison between SNP array and qRT-PCR for NOS1 and EDN1 SNPs. Click here for file
