# Supplementary material for: Genetic variance in Nitric Oxide Synthase and Endothelin Genes among children with and without Endothelial Dysfunction
Source: J Transl Med. 2013 Sep 25;11:227. doi: 10.1186/1479-5876-11-227 (PMC3849009; doi:10.1186/1479-5876-11-227)
Supplement: Additional file 1: Table S1 — List of single nucleotide polymorphisms (SNPs) of NOS1 and EDN1 genes. [file 1479-5876-11-227-S1.docx]

**Additional file 1: Table S1 List of single nucleotide polymorphisms (SNPs) of NOS1 and EDN1 genes.**

| Gene | SNP  number | Chr. | Name | Alleles | Chr. position | Location | MAF% | SNP type |
| --- | --- | --- | --- | --- | --- | --- | --- | --- |
| NOS1 | 1 | 12 | rs6490121 | A/G | 117708195 | Intron | 38 | Transition substitution |
|  | 2 | 12 | rs2293052 | C/T | 117715620 | Intron | 25 | Transition substitution |
|  | 3 | 12 | rs3825102 | A/C | 117720185 | Intron | 28 | Transversion substitution |
|  | 4 | 12 | rs4767529 | C/G | 117721812 | Intron | 33 | Transversion substitution |
|  | 5 | 12 | rs561712 | A/G | 117752069 | Intron | 34 | Transition substitution |
|  | 6 | 12 | rs549098 | C/T | 117754327 | Intron | 33 | Transition substitution |
|  | 7 | 12 | rs567581 | A/G | 117757512 | Intron | 33 | Transition substitution |
|  | 8 | 12 | rs483589 | A/G | 117781531 | Intron | 32 | Transition substitution |
|  | 9 | 12 | rs693534 | A/G | 117784718 | Intron | 32 | Transition substitution |
|  | 10 | 12 | rs9658255 | C/G | 117799756 | NA | 33 | NA |
|  | 11 | 12 | rs1879417 | C/T | 117803515 | Intergenic | 45 | Transition substitution |
| EDN1 | 1 | 6 | rs1476046 | A/G | 12293221 | Intron | 21 | Transition substitution |
|  | 2 | 6 | rs4714384 | C/T | 12297853 | Intergenic | 49 | Transition substitution |

NA, data not available.
