# Supplementary material for: Genetic variance in Nitric Oxide Synthase and Endothelin Genes among children with and without Endothelial Dysfunction
Source: J Transl Med. 2013 Sep 25;11:227. doi: 10.1186/1479-5876-11-227 (PMC3849009; doi:10.1186/1479-5876-11-227)
Supplement: Additional file 2: Table S2 — Distributions of allele and genotype frequencies of NOS1 and EDN1 SNPs in children with and without endothelial dysfunction compare between array and real-time PCR. [file 1479-5876-11-227-S2.docx]

**Additional file 2: Table S2 Distributions of allele and genotype frequencies of NOS1 and EDN1 SNPs in children with and without endothelial dysfunction compare between array and real-time PCR.**

| No. | Gene | SNP | Methods |  | NEF | | ED | | *P*-value |
| --- | --- | --- | --- | --- | --- | --- | --- | --- | --- |
| 1 | NOS1 | rs3825102 | Array | Allele | n=77 | | n=32 | |  |
|  |  |  |  | A/C | n | % | n | % |  |
|  |  |  |  | AA | 9 | 12 | 2 | 6 | 0.004 |
|  |  |  |  | AC | 20 | 26 | 19 | 60 |  |
|  |  |  |  | CC | 48 | 62 | 11 | 34 |  |
|  |  |  |  | Allele A | 38 | 25 | 23 | 36 |  |
|  |  |  |  | Allele C | 116 | 75 | 41 | 64 |  |
|  |  |  | Real-time |  | NEF | | ED | | *P*-value |
|  |  |  | PCR | Allele | n=77 | | n=32 | |  |
|  |  |  |  | A/C | n | % | n | % |  |
|  |  |  |  | AA | 6 | 8 | 4 | 13 | 0.283 |
|  |  |  |  | AC | 32 | 41 | 17 | 53 |  |
|  |  |  |  | CC | 39 | 51 | 11 | 34 |  |
|  |  |  |  | Allele A | 44 | 29 | 25 | 39 |  |
|  |  |  |  | Allele C | 110 | 71 | 39 | 61 |  |
| 2 | NOS1 | rs483589 | Array |  | NEF | | ED | | *P*-value |
|  |  |  |  | Allele | n=77 | | n=32 | |  |
|  |  |  |  | A/G | n | % | n | % |  |
|  |  |  |  | AA | 10 | 13 | 11 | 34 | 0.014 |
|  |  |  |  | AG | 29 | 38 | 13 | 41 |  |
|  |  |  |  | GG | 38 | 49 | 8 | 25 |  |
|  |  |  |  | Allele A | 49 | 32 | 35 | 55 |  |
|  |  |  |  | Allele G | 105 | 68 | 29 | 45 |  |
|  |  |  | Real-time |  | NEF | | ED | | *P*-value |
|  |  |  | PCR | Allele | n=77 | | n=32 | |  |
|  |  |  |  | A/G | n | % | n | % |  |
|  |  |  |  | AA | 12 | 16 | 7 | 22 | 0.687 |
|  |  |  |  | AG | 31 | 40 | 13 | 41 |  |
|  |  |  |  | GG | 34 | 44 | 12 | 37 |  |
|  |  |  |  | Allele A | 55 | 36 | 27 | 42 |  |
|  |  |  |  | Allele G | 99 | 64 | 37 | 58 |  |

| 3 | EDN1 | rs1476046 | Array |  | NEF | | ED | | *P*-value |
| --- | --- | --- | --- | --- | --- | --- | --- | --- | --- |
|  |  |  |  | Allele | n=77 | | n=32 | |  |
|  |  |  |  | A/G | n | % | n | % |  |
|  |  |  |  | AA | 2 | 2 | 0 | 0 | 0.004 |
|  |  |  |  | AG | 39 | 51 | 6 | 19 |  |
|  |  |  |  | GG | 36 | 47 | 26 | 81 |  |
|  |  |  |  | Allele A | 43 | 28 | 6 | 9 |  |
|  |  |  |  | Allele G | 111 | 72 | 58 | 91 |  |
|  |  |  | Real-time |  | NEF | | ED | | *P*-value |
|  |  |  | PCR | Allele | n=77 | | n=32 | |  |
|  |  |  |  | A/G | n | % | n | % |  |
|  |  |  |  | AA | 2 | 2 | 1 | 3 | 0.263 |
|  |  |  |  | AG | 32 | 42 | 8 | 25 |  |
|  |  |  |  | GG | 43 | 56 | 23 | 72 |  |
|  |  |  |  | Allele A | 36 | 23 | 10 | 16 |  |
|  |  |  |  | Allele G | 118 | 77 | 54 | 84 |  |
| 4 | EDN1 | rs4714384 | Array |  | NEF | | ED | | *P*-value |
|  |  |  |  | Allele | n=77 | | n=32 | |  |
|  |  |  |  | C/T | n | % | n | % |  |
|  |  |  |  | CC | 21 | 27 | 2 | 6 | 0.018 |
|  |  |  |  | CT | 40 | 52 | 17 | 53 |  |
|  |  |  |  | TT | 16 | 21 | 13 | 41 |  |
|  |  |  |  | Allele C | 82 | 53 | 21 | 33 |  |
|  |  |  |  | Allele T | 72 | 47 | 43 | 67 |  |
|  |  |  | Real-time |  | NEF | | ED | | *P*-value |
|  |  |  | PCR | Allele | n=77 | | n=32 | |  |
|  |  |  |  | C/T | n | % | n | % |  |
|  |  |  |  | CC | 11 | 14 | 8 | 25 | 0.344 |
|  |  |  |  | CT | 43 | 56 | 14 | 44 |  |
|  |  |  |  | TT | 23 | 30 | 10 | 31 |  |
|  |  |  |  | Allele C | 65 | 42 | 30 | 47 |  |
|  |  |  |  | Allele T | 89 | 58 | 34 | 53 |  |
