# Supplementary material for: Genetic variance in Nitric Oxide Synthase and Endothelin Genes among children with and without Endothelial Dysfunction
Source: J Transl Med. 2013 Sep 25;11:227. doi: 10.1186/1479-5876-11-227 (PMC3849009; doi:10.1186/1479-5876-11-227)
Supplement: Additional file 3: Table S3 — Comparison between SNP array and qRT-PCR for NOS1 and EDN1 SNPs. [file 1479-5876-11-227-S3.docx]

**Additional file 3: Table S3 Comparison between SNP array and** **qRT-PCR for NOS1 and EDN1 SNPs.**

| Group | No. | NOS1 gene | | | | EDN1 gene | | | |
| --- | --- | --- | --- | --- | --- | --- | --- | --- | --- |
|  |  | rs3825102 (A/C) | | rs483589 (A/G) | | rs1476046 (A/G) | | rs4714384 (C/T) | |
|  |  | Array | Real-time | Array | Real-time | Array | Real-time | Array | Real-time |
| NEF=77 | 1 | AC | AC | GG | GG | AG | AG | CC | CC |
|  | 2 | CC | AC | GG | GG | AG | AG | CT | CT |
|  | 3 | AA | AA | AA | AA | GG | GG | CT | CT |
|  | 4 | AA | AA | AA | AA | GG | GG | CT | CT |
|  | 5 | CC | CC | AG | AG | GG | AG | CC | CT |
|  | 6 | AA | CC | AG | GG | AG | AG | CT | CT |
|  | 7 | AC | CC | GG | AG | AG | AG | CT | CT |
|  | 8 | AC | AC | AA | GG | GG | GG | CT | CT |
|  | 9 | CC | AC | AG | AA | AG | GG | CT | CT |
|  | 10 | AC | CC | AG | AG | AG | AG | CC | CC |
|  | 11 | AC | AC | AG | AG | GG | GG | TT | TT |
|  | 12 | CC | AC | AG | AG | GG | GG | CC | CC |
|  | 13 | CC | AC | GG | AG | AA | AA | TT | TT |
|  | 14 | AC | CC | AG | GG | AG | AG | CT | CT |
|  | 15 | CC | CC | GG | GG | GG | GG | CT | CT |
|  | 16 | CC | AC | AA | AG | AG | AG | CT | CT |
|  | 17 | CC | CC | GG | AG | GG | GG | TT | TT |
|  | 18 | AA | AC | AA | AG | AG | GG | CC | TT |
|  | 19 | AA | AC | AA | AA | AG | GG | TT | CT |
|  | 20 | AA | AC | AG | AG | GG | AG | CC | TT |
|  | 21 | AA | AA | AA | AA | GG | GG | CT | CT |
|  | 22 | CC | AC | GG | GG | AG | GG | TT | CT |
|  | 23 | CC | CC | AA | AA | GG | GG | CT | CT |
|  | 24 | AC | AC | AG | AG | AG | GG | TT | TT |
|  | 25 | CC | CC | GG | GG | GG | AG | CT | TT |
|  | 26 | AC | AC | AG | AG | GG | GG | CC | CT |
|  | 27 | CC | CC | GG | GG | GG | GG | CT | TT |
|  | 28 | CC | CC | GG | GG | AG | GG | CT | CT |
|  | 29 | CC | CC | GG | GG | GG | GG | CT | CT |
|  | 30 | CC | CC | GG | GG | AG | AG | CT | CT |
|  | 31 | CC | CC | GG | GG | GG | AG | CT | CT |
|  | 32 | AC | AC | AG | AG | AG | GG | TT | CT |
|  | 33 | CC | AC | GG | GG | AG | GG | CT | TT |
|  | 34 | CC | AC | GG | AA | AG | AG | CC | TT |
|  | 35 | AC | AC | AG | AG | AG | GG | CT | CT |
|  | 36 | CC | CC | AG | GG | GG | GG | CC | TT |
|  | 37 | AC | CC | AG | AG | GG | AG | CT | TT |
|  | 38 | CC | CC | GG | AA | AG | GG | TT | CC |
|  | 39 | CC | AA | AG | AA | AG | GG | CC | CT |
|  | 40 | CC | AC | GG | AG | GG | AG | CT | TT |
|  | 41 | AC | CC | AG | AG | GG | GG | CT | TT |
|  | 42 | CC | CC | GG | GG | GG | GG | CC | CT |
|  | 43 | CC | AC | GG | AG | GG | GG | CT | CT |
|  | 44 | CC | CC | GG | AA | GG | GG | CT | CT |
|  | 45 | CC | CC | GG | GG | AG | GG | CT | TT |
|  | 46 | CC | CC | AG | GG | AG | GG | CT | CC |
|  | 47 | AC | AC | GG | AG | AG | GG | CT | CT |
|  | 48 | AC | AC | AG | AG | GG | GG | TT | CT |
|  | 49 | CC | CC | GG | GG | AG | AG | CC | CT |
|  | 50 | AC | AC | AG | AG | AG | AG | TT | CT |
|  | 51 | CC | AC | AG | AG | AG | AG | CT | TT |
|  | 52 | CC | CC | GG | AG | GG | AG | CT | CT |
|  | 53 | AC | CC | AG | GG | GG | GG | CT | CT |
|  | 54 | CC | AC | GG | AG | AG | GG | CT | CT |
|  | 55 | AC | CC | AG | GG | AG | AG | CT | CT |
|  | 56 | CC | AC | GG | AG | AA | AG | CT | CT |
|  | 57 | CC | CC | GG | GG | GG | GG | CT | CT |
|  | 58 | CC | AC | AG | AG | AG | GG | CC | CT |
|  | 59 | CC | AC | GG | AG | GG | GG | CC | TT |
|  | 60 | CC | AC | GG | GG | GG | GG | CT | TT |
|  | 61 | CC | CC | AG | GG | AG | GG | CT | CT |
|  | 62 | CC | CC | GG | AG | AG | AG | TT | CT |
|  | 63 | CC | CC | GG | GG | GG | AG | TT | TT |
|  | 64 | AC | CC | AG | GG | GG | GG | CT | TT |
|  | 65 | AA | CC | AA | GG | AG | GG | CT | CT |
|  | 66 | CC | AA | GG | AA | GG | AG | TT | CT |
|  | 67 | CC | CC | GG | GG | AG | AG | TT | TT |
|  | 68 | CC | CC | GG | GG | AG | AG | CC | TT |
|  | 69 | AA | CC | AA | GG | AG | AG | CC | CC |
|  | 70 | CC | AA | GG | AA | AG | AG | CC | CC |
|  | 71 | CC | CC | GG | GG | AG | AG | CC | CC |
|  | 72 | CC | CC | AG | GG | AG | AG | CC | CC |
|  | 73 | CC | AC | GG | AG | GG | AG | CC | TT |
|  | 74 | CC | AC | AG | AG | GG | GG | TT | CC |
|  | 75 | CC | CC | GG | GG | AG | AA | CT | CT |
|  | 76 | AC | CC | AG | GG | GG | AG | CC | CT |
|  | 77 | AC | AC | AG | AG | GG | GG | TT | CC |

| ED=32 | 1 | AC | AC | AA | AA | GG | GG | TT | TT |
| --- | --- | --- | --- | --- | --- | --- | --- | --- | --- |
|  | 2 | AC | AC | AA | AA | GG | GG | TT | TT |
|  | 3 | AC | AC | AA | GG | GG | GG | TT | CT |
|  | 4 | AC | AC | GG | GG | GG | GG | CC | CC |
|  | 5 | AC | AC | AG | AG | GG | GG | CC | TT |
|  | 6 | AC | AC | AA | AG | GG | GG | TT | CC |
|  | 7 | CC | CC | AG | GG | GG | GG | CT | CC |
|  | 8 | AC | AA | AG | AG | GG | AG | CT | CC |
|  | 9 | AC | AC | AG | AG | GG | GG | CT | CC |
|  | 10 | AC | AC | AA | AA | GG | GG | TT | CT |
|  | 11 | CC | AC | AG | AG | GG | AG | CT | TT |
|  | 12 | AC | AC | AG | AG | GG | GG | TT | TT |
|  | 13 | CC | CC | GG | AG | GG | GG | TT | CT |
|  | 14 | CC | AC | AA | AG | GG | GG | CT | CC |
|  | 15 | CC | AC | GG | AG | GG | GG | CT | CT |
|  | 16 | AC | AA | AA | AA | GG | GG | CT | CT |
|  | 17 | CC | CC | AA | GG | GG | GG | TT | CT |
|  | 18 | AA | CC | AA | GG | AG | GG | CT | CT |
|  | 19 | AC | CC | GG | GG | AG | GG | CT | TT |
|  | 20 | AC | AC | AG | AG | GG | AG | TT | CT |
|  | 21 | AA | CC | AA | AG | AG | AG | TT | CC |
|  | 22 | AC | CC | AG | GG | GG | AG | TT | TT |
|  | 23 | AC | AA | AG | AA | GG | GG | CT | TT |
|  | 24 | AC | AC | AG | GG | GG | GG | TT | CT |
|  | 25 | AC | AC | AG | AG | GG | AG | CT | CT |
|  | 26 | CC | AA | GG | AA | GG | AG | CT | TT |
|  | 27 | AC | CC | AA | AG | AG | GG | CT | CC |
|  | 28 | CC | AC | AG | GG | AG | AG | CT | CT |
|  | 29 | AC | CC | AG | GG | GG | AA | CT | CT |
|  | 30 | CC | AC | GG | AA | GG | GG | CT | CT |
|  | 31 | CC | CC | GG | GG | GG | GG | CT | TT |
|  | 32 | CC | CC | GG | GG | AG | GG | TT | CT |
